# Chemotypic diversity of the fungus *Epichloë brachyelytri* symbiotic with the wild forest grass *Brachyelytrum erectum*

**DOI:** 10.1128/aem.00754-26

**Published:** 2026-08-14

**Authors:** Padmaja Nagabhyru, Simona Florea, Huazhen Liu, Pradeep Kachroo, Patrick J. Calie, Carolyn A. Young, Christopher L. Schardl

**Affiliations:** 1 Department of Plant Pathology, University of Kentucky 242854, Lexington, Kentucky, USA; 2 Department of Biological Sciences, Eastern Kentucky University 167100, Richmond, Kentucky, USA; 3 Department of Entomology and Plant Pathology, North Carolina State University 169129, Raleigh, North Carolina, USA; Royal Botanic Gardens Kew, Surrey, United Kingdom

**Keywords:** alkaloids, chemotypic diversity, *Epichloë*, molecular evolution, symbiosis

## Abstract

Many cool-season grasses (Poaceae subfam. Poöideae) host seed-transmissible symbionts (endophytes) in the fungal genus *Epichloë*, which can produce diverse alkaloids that protect against invertebrate and, in some cases, vertebrate herbivores. Rarely have population surveys been conducted to assess comprehensive alkaloid profiles and diversity of *Epichloë* in wild grasses. In this study, we surveyed *Brachyelytrum erectum*, which is a woodland grass in an early-diverging lineage of Poöideae, and commonly symbiotic with *Epichloë brachyelytri*. Analytical methods based on high-resolution UHPLC-MS/MS were refined to provide rapid, comprehensive detection, and quantitation of *E. brachyelytri* alkaloids, for six *B. erectum* populations in Kentucky. Chemotypes were identified with two or three of the alkaloids *exo*-1-acetamidopyrrolizidine (**1**), chanoclavine (**2**), and peramine (**3**). Both **1** and **2** are known as intermediates in biosynthetic pathways to more complex alkaloids, and chemotypes having both **1** and **2** as pathway end-products are novel. Such chemotypes were also identified in other species, and phylogenetic analysis indicated their multiple origins by a combination of convergent evolution, possible horizontal gene transfer, and interspecific hybridization. Alkaloid levels were comparable between most populations and at most plant developmental stages. Levels of **3** were compared between *E. brachyelytri* variants with and without **1**, providing evidence for competition between the pathways in young shoots, but not in older leaves or seeds. Furthermore, levels of **1** and **2** were moderate to high compared with their respective alkaloid classes in other grass-*Epichloë* symbiotic systems. We conclude that production of the alkaloids likely represents an important metabolic investment by *E. brachyelytri*.

**IMPORTANCE:**

Defensive mutualisms, symbioses of hosts with organisms that defend them against parasites or predators, play important ecological roles. A widespread example is protection of cool-season grasses by symbiotic *Epichloë* species, which are fungi that transmit in seeds and produce several kinds of anti-insect alkaloids. Profiles of alkaloids evolve due to shifting balances of their benefits and the costs of producing them. In this study, *Epichloë brachyelytri* symbiotic with the wild forest grass *Brachyelytrum erectum* produced three alkaloids, of which two have been rarely reported. Furthermore, variations in its alkaloid profiles and quantities of each alkaloid at different plant growth stages and tissues suggested that occasional loss of its most abundant alkaloid can be adaptive due to the metabolic load of producing it. Although rare, similar alkaloid profiles were identified in several other species in which they arose by a combination of convergent evolution, possible horizontal gene transfer, and interspecific hybridization.

## INTRODUCTION

**Fig 1:**
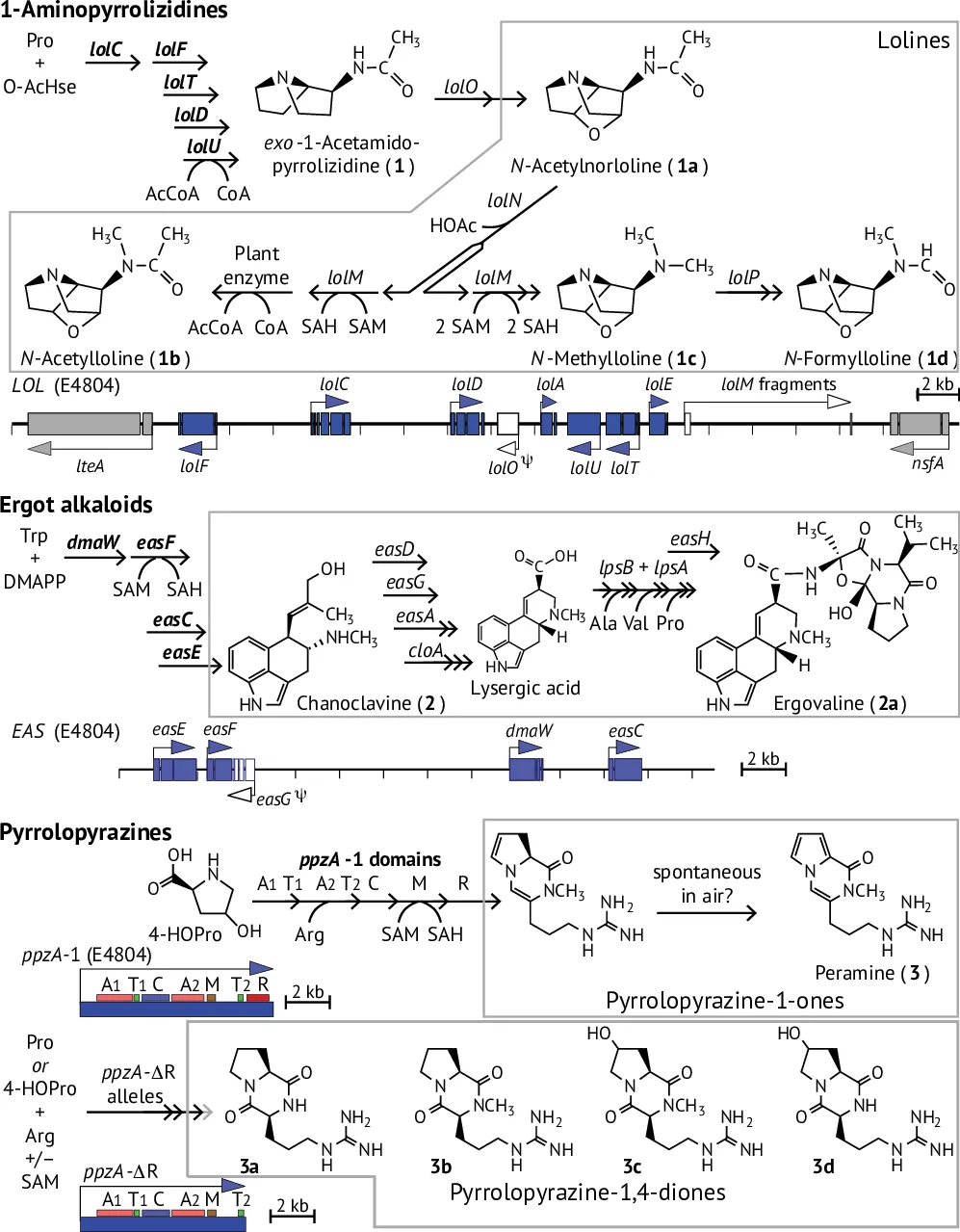
Alkaloid structures and *Epichloë* genes required for their biosynthesis. Pathways for 1-aminopyrrolizidines, ergot alkaloids, and pyrrolopyrazines in many *Epichloë* species are indicated with genes encoding enzymes listed next to the arrows, and those found in *E. brachyelytri* E4804 are shown in bold text. Multiple arrowheads indicate multiple successive steps carried out by the same enzyme (LolO, LolM, LolP, EasA, or CloA) or by multiple enzymatic domains of the same polypeptide (LspA or PpzA-1). Standard abbreviations designate l-amino acids, and other abbreviations are: 4-HOPro = 4-hydroxyproline, O-AcHse = *O*-acetylhomoserine, HOAc = acetic acid, SAM = *S*-adenosylmethionine, SAH = *S*-adenosylhomocysteine, AcCoA = acetyl coenzyme A, CoA = coenzyme A, DMAPP = dimethylallyl diphosphate, but ATP and redox cosubstrates and their products are not shown. Maps of genes and gene clusters in *E. brachyelytri* E4804 for the 1-aminopyrrolizidine (*LOL*), ergot alkaloid (*EAS*), and pyrrolopyrazine (*ppzA*) pathways are shown, as is the variant *ppzA*-∆R allele in some *Epichloë* species, such as in *E. siegelii*.

*Epichloë* species (fungal order Hypocreales, family Clavicipitaceae) are systemic symbionts of cool-season grasses (Poaceae, subfam. Poöideae), and almost all strains of *Epichloë* have genes for bioprotective alkaloids in up to four chemical classes (1) (Fig. 1). The biosynthetic steps to ergot alkaloids are specified by a cluster of genes in the *EAS* locus (reviewed in reference 2), steps to indole-diterpenes are specified by the *IDT* cluster (3, 4), steps to 1-aminopyrrolizidines are specified by the *LOL* cluster (5–8), and steps to pyrrolopyrazines are specified by domains of the *ppzA* gene (formerly *perA*) (9).

Historically, major species of these four alkaloid classes (Fig. 1) were discovered and linked to endophytic *Epichloë* species in research aimed at identifying causes of livestock toxicoses associated with certain cool-season pasture grasses. First, the ergot alkaloid ergovaline was implicated in the symptoms of fescue toxicosis (“summer slump” and “fescue foot”) as well as reproductive failures in animals grazing *Lolium arundinaceum* (tall fescue) (10, 11). Then, the lolitrems, a group of complex indole-diterpenes, were implicated in ryegrass staggers observed in animals grazing *Lolium perenne* (perennial ryegrass) (12, 13). Similar investigations also identified two novel alkaloid classes, the saturated 1-aminopyrrolizidines called lolines (14) and the pyrrolopyrazine alkaloid peramine (15). Ultimately, both lolines (reviewed in reference 16) and peramine (17) were found to be protective of the grasses against insects, but not appreciably toxic to mammals. Lolines have broad-spectrum anti-insect activity (18–20) (reviewed in reference 16) and can also affect nematodes (21), and peramine is an important deterrent of insects (22).

The bioprotective effects of *Epichloë* species and their alkaloids in agriculture have been recognized for decades as agriculturally relevant (10, 23, 24), but related symbiotic systems in the wild have received less attention and have even been downplayed as potential mutualisms (25). Unfortunately, the vast majority of ecological studies have only used targeted analysis of a subset of the epichloid alkaloids known 30 years ago, let alone the substantially greater variety discovered since. Perhaps the main reason for this deficiency has been the laborious, specialized, and expensive techniques previously required to analyze each alkaloid. No longer should this be a limitation given current knowledge of the pathways and the underlying genetics (reviewed in reference 26), coupled with recent advances in small-molecule analytical techniques and instrumentation to facilitate comprehensive studies of wild populations.

Variation in alkaloid profiles (chemotypic diversity) is found between species, and even between and within populations, of *Epichloë*. For example, in both *Epichloë bromicola* and *Epichloë festucae,* isolates have been identified with genes for different 1-aminopyrrolizidines, different pyrrolopyrazines, different indole-diterpenes, and the ergot alkaloid ergovaline. Their chemotypes comprise one, or various combinations of 2–3 of these four alkaloid classes (1, 27, 28). Other dimensions of diversity in *Epichloë* species and strains, which can be related to their chemotypic diversity (1, 29), include their host ranges, relative horizontal or vertical transmission, relative asexual or sexual reproduction, genome ploidy (30, 31), and different physiological and morphological effects on their hosts (32).

Some chemotypic diversification in haploid *Epichloë* species results from sexual recombination. The *Epichloë* sexual cycle starts with emergence of stromata (singular, *stroma*), which produce spermatia and ascogonia (33), and requires cross-fertilization between opposite mating types (34) designated *MTA* and *MTB* (35). Transfer of spermatia between stromata (“pollination”) is typically facilitated by *Epichloë-*specialized fly species in genus *Botanophila* whose larvae develop on the maturing stromata (36, 37). Lowered alkaloid levels in stromata may allow for this additional mutualistic interaction between fungus and insect (38). However, the *Epichloë* sexual cycle requires that both mating types be present and manifest stromata, conditions that are not always met. In populations of many grasses, including (but not limited to) *Brachyelytrum erectum, Elymus* species, North American *Agrostis* and *Poa* species, and many *Lolium* and *Festuca* species, *Epichloë* stroma production is rare (1, 27, 39–41). Therefore, many *Epichloë* strains rely mainly or exclusively on clonal propagation by colonizing host seeds. Because the symbioses are systemic in host grasses, they maintain a benign association with host inflorescence primordia, ovaries and eventually the embryo, ensuring that they are transmitted vertically from mother to daughter plants (42–44). Such reliance on vertical transmission is predicted to select for abundant or diverse alkaloid production (26).

Plant hosts of *Epichloë* species represent a broad taxonomic range of the Poöideae (reviewed in reference 45). Several species occur in important pasture, meadow and range grasses, as well as crop wild relatives, in tribes Poeae, Triticeae, and Bromeae (1, 46, 47). Tribes Stipeae and Meliceae, which are relatively basal to those (48), also include hosts of *Epichloë* species (1, 49–51). Tribe Brachyelytreae has been identified as splitting basally from the rest of subfam. Poöideae (48) and comprises the woodland grasses *B. erectum* (bearded shorthusk) and *Brachyelytrum aristosum* (northern shorthusk) in North America, as well as *Brachyelytrum japonicum* in East Asia (52). The endophyte *E. brachyelytri* is commonly symbiotic with *B. erectum*, in which it is transmitted vertically in seeds, and probably also horizontally transmitted occasionally via sexually-derived spores (53). Genetic variation for alkaloids may occur by mutations, which would most often involve gene loss or inactivation, as well as sexual recombination.

Natural chemotypic variation has allowed for selection of endophyte chemotypes suitable for forage and pasture grass cultivars (24). Furthermore, considerable quantitative variation in *Epichloë* alkaloids has been documented in cultivated plants, and in some symbioses their levels may be so high that their production imposes a significant metabolic burden on the host plants (54, 55). Quantitative studies of these alkaloids in wild grass populations are rare, and none addresses the diversity of alkaloids now known to comprise each class. Here, we employ ultra-high performance liquid chromatography qTOF tandem mass spectrometry (UHPLC-MS/MS) to show that strains of *E. brachyelytri* produce **1**, **2** and **3** (Fig. 1) *in planta* at levels that are moderate to high compared with other grass-*Epichloë* holobionts, and that some populations of *E. brachyelytri* exhibit chemotypic variation. Quantitative profiles of chemotypic variants within populations suggest that the pathways to **1** and **3** may compete with each other in young plant shoots, suggesting that alkaloid production may be a significant metabolic burden on the *B. erectum-E. brachyelytri* symbiosis.

## RESULTS

### Chemotypic diversity of *Epichloë brachyelytri* among and within populations

Samples from five natural *B. erectum* populations and one planted population were analyzed by PCR for *E. brachyelytri* genes *mtAC* (for mating type *MTA*) and *mtBA* (for *MTB*) to estimate percentages of endophyte-symbiotic (E+) and asymbiotic (E–) plants, and for an initial indication of genetic diversity of *E. brachyelytri* (Table 1). Endophyte abundance ranged from 42% to 94% E+ plants. All samples from the MACAV and KRPTD populations were *MTB*. The FLCLF, RRGSC, and CACAV populations had individuals of both mating types, *MTA* and *MTB*.

**TABLE 1:** Surveyed Kentucky populations of *Brachyelytrum erectum*, with endophyte status and genotypes

Popn^*a*^	Tissue	GPS	Date collected	E+%^*b*^	Genotypes^*c*^				
					*MTA LOL EAS ppzA*-1	*MTB LOL EAS ppzA*-1	*MTA EAS ppzA*-1	*MTB EAS ppzA*-1	*MTBLOL ppzA*-1
CACAV	Leaves	38.3730 N, 83.1240 W	8 Jun 2024	42	10	7	0	0	7
FLCLF	Leaves, seeds	37.9025 N, 84.3618 W	25 Aug 2022	87	0	0	15	5	0
KRPTD	Leaves, seeds	37.7656 N, 84.6224 W	11 Oct 2021	57	0	30	0	20	0
KYARB^*d*^	Shoots	38.0140 N, 84.5075 W	26 Apr 2025	94	0	16	0	0	0
MACAV	Leaves, seeds	37.1837 N, 86.0997 W	29 Aug 2021	79	0	38	0	0	0
RRGSC	Leaves, seeds	37.8038 N, 83.5903 W	20 Aug 2022	58	9	5	2	18	0
RRGSC	Shoots	37.8038 N, 83.5903 W	17 April 2025	50	0	6	1	6	0

^*a*^ Populations are designated as follows: CACAV = Carter Caves State Resort Park, FLCLF = Floracliff Nature Sanctuary, KRPTD = Kentucky River Palisades Tom Dorman State Nature Preserve, KYARB = Kentucky Arboretum, MACAV = Mammoth Cave National Park; RRGSC = Red River Gorge Geological Area near Swift Camp Creek.

^*b*^ Percent of plants positive for *Epichloë brachyelytri* in the *B. erectum* population.

^*c*^ Numbers of each *Epichloë brachyelytri* genotype identified by analysis of *Brachyelytrum erectum* plants or endophyte cultures from each location. Genes for mating-type idiomorph *MTA* or *MTB*, and the presence of genes for determinant steps in biosynthesis of 1-aminopyrrolizidines (*LOL*), ergot alkaloids (*EAS*), and peramine (*ppzA*-1), are indicated. All were negative for *idtG* for the determinant step in indole-diterpene biosynthesis.

^*d*^ KYAb plants were grown from seeds collected in Lewis Co., KY, at 38.6472 N, 83.5726 W.

For E+ plant samples, the presence of each alkaloid locus was assessed by PCR targeting *lolC* in the *LOL* cluster, *dmaW* in the *EAS* cluster, *idtG* in the *IDT* cluster, and the T2 and R domains of the multi-domain *ppzA*-1 gene (Fig. 1). None of the samples were positive for *idtG*, but most were positive for each of the other alkaloid loci. All samples that were positive for the *ppzA*-1 T2 domain were also positive for the reductase (R) domain that is required for biosynthesis of **3** instead of pyrrolopyrazine-1,4-diones **3a–d** (Fig. 1).

Variation for presence or absence (+/– polymorphism) of the determinant alkaloid genes was evident among and within populations, whereby all endophytes from MACAV and KYARB tested positive for *lolC, dmaW,* and *ppzA*-1, all endophytes from FLCLF tested positive for *dmaW* and *ppzA*-1 but not *lolC*, samples from the KRPTD and RRGSC populations tested positive for *dmaW* and *ppzA*-1 but were +/– polymorphic for *lolC*, and samples from CACAV had *lolC* and *ppzA*-1 but were +/– polymorphic for *dmaW* (Table 1).

### Alkaloid levels in *Brachyelytrum erectum*

The published genome (GenBank accession GCA_000222915.1) and alkaloid gene sequences of *E. brachyelytri* isolate E4804 (Table 2; Fig. 1) indicate an apparently complete *ppzA*-1 gene, but the only potentially functional genes at the other alkaloid loci are for the five steps to **1** (*LOL* cluster), and the four initial steps to **2** (*EAS* cluster). Alkaloid profiles (Table 3) confirmed those predicted end-products in all *B. erectum* samples that were positive for the respective alkaloid loci. Furthermore, except for four outliers, samples with each alkaloid locus had comparable levels of that alkaloid. Two outliers were among the *EAS+* leaf samples from CACAV, of which one had only a trace of **2** and the other had only a trace of **3**. The other two outliers were among the *LOL–* samples from RRGSC, and had levels of both **2** and **3** in leaves that were less than 10% those of the other leaf samples from that location.

**TABLE 2:** *Atkinsonella* and *Epichloë* strains and sequence accessions^*a*^

Species^*b*^	Strain	*tefA*	*LOL*	*EAS*	*ppzA*-1	*ppzA*-∆R
*A. hypoxylon*	B4728	KP689546	KF056806	PZ260826	–	–
*E. amarillans*	E57	KP689562	JF830812– JF830813	–	PZ260827	–
*E. amarillans*	E4668	KP689563	KC990436	KC989564	MN605952	–
*E. aotearoae*	E899	MF928034	KC990449– KC990451	–	ψ^*c*^	–
*E. brachyelytri*	E4804	KP689564	JF800659–JF800661	JN177507– JN177508	JN613323	–
*E. bromicola*	AL0426/2	KP689559	PP187353	–	–	KP347846ψ
*E. bromicola*	E6357-1	PP002260	PP187031	–	PZ260828	–
*E. bromicola*	E7626	MF838712	–	MF838703– MF838706	MF838705	–
*E. calamagrostidis*	ATCC 200745	KP689561	KC990439– KC990440	–	KP347874	–
*E. canadensis* (a)	CWR 34	PP002257	PP187337– PP187338	–	PZ260833ψ	–
*E. canadensis* (a)	E4815	PP438472	KC990444– KC990447	KC989567– KC989568	unassembled	–
*E. canadensis* (e)	CWR 34	PP002258	–	PZ260820– PZ260822	PZ260832ψ	–
*E. canadensis* (e)	E4815	PP438477	–	KC989565, KC989612	unassembled	–
*E. clarkii*	E422	PP002259	–	PZ260823–PZ260825	ψ	–
*E. elymi*	E56	KP689557	–	JX439640– JX439642	PZ260829	–
*E. festucae*	E2368	MF928029	JF830815, EF012267	JN167225–JN167226	–	JN640287
*E. festucae*	Fl1	MF928031	–	JN177500–JN177501	AB205145	–
*E. glyceriae*	E277	KP689560	JF800663– JF800665	JN177503–JN177504	–	–
*E. mollis*	AL9924	KP689567	–	KC989572–KC989573	KP347873	–
*E. poae*	E5819	KP689553	–	JN182230–JN182232	–	JN640289
*E. siegelii* (f)	E915(f)	AF308133	–	–	–	KP719971
*E. siegelii* (o)	E915(o)	AF308132	PP187331	–	KP719972ψ	–
*E. stromatolonga*	Cnja206	OP654686	PP187349– PP187351	–	PZ260830	–
*E. typhina*	E5859	OP654698	PP187345– PP187347	–	PZ260831	–
*E. uncinata* (o)	E167(o)	OP654702	AY723749	–	ψ	–
*E. uncinata* (p)	E167(p)	OP654701	AY724686, AY723750	–	ψ	–

^*a*^ Accessions are in the GenBank database of the National Center for Biotechnology Information. A minus sign (–) indicates that the sequence is absent in that isolate.

^*b*^ Letters in parentheses indicate ancestral genomes *E. amarillans* (a), *E. elymi* (e), *E. festucae* (f), *E. bromicola* (o)*, *and *E. poae* (p) in the interspecific hybrids.

^*c*^ ψ = pseudogene.

**TABLE 3:** *Epichloë* alkaloid quantities in plant tissues^*a*^

Plant sp. and population or accession	*Epichloë* sp. and strain	Pred. chem.^*b*^	*n*	Alkaloid (µg/g dry wt ± SD)^*c*^						
				1	1a	1b–d	2	2a	3	3b
Young shoots										
*Brachyelytrum erectum* KYARB	*E. brachyelytri*	**1, 2, 3**	6	331 ± 86	–	–	175 ± 47	–	14.8 ± 3.6	–
*B. erectum* RRGSC	*E. brachyelytri*	**1, 2, 3**	4	1,121 ± 606	–	–	**123 ± 13**	–	**8.2 ± 1.9**	–
*B. erectum* RRGSC	*E. brachyelytri*	**2, 3**	5	–	–	–	**83 ± 18**	–	**11.1 ± 1.7**	–
Leaves										
*B. erectum* CACAV^*d*^	*E. brachyelytri*	**1, 2, 3**	6	1,998 ± 682	–	–	154 ± 44	–	10.7 ± 3.5	–
*B. erectum* CACAV	*E. brachyelytri*	**1, 3**	3	1,958 ± 1,191	–	–	–	–	11.8 ± 6.5	–
*B. erectum* CACAV^*e*^	*E. brachyelytri*	**1, 2, 3**	1	1,351	–	–	tr	–	8.0	–
*B. erectum* CACAV^*e*^	*E. brachyelytri*	**1, 2, 3**	1	2,127	–	–	112	–	tr	–
*B. erectum* KRPTD	*E. brachyelytri*	**1, 2, 3**	6	1,978 ± 441	–	–	**122 ± 21**	–	**8.8 ± 1.5**	–
*B. erectum* KRPTD	*E. brachyelytri*	**2, 3**	6	–	–	–	**78 ± 13**	–	**6.1 ± 1.4**	–
*B. erectum* MACAV	*E. brachyelytri*	**1, 2, 3**	7	3,227 ± 914	–	–	167 ± 34	–	12.5 ± 3.6	–
*B. erectum* RRGSC	*E. brachyelytri*	**1, 2, 3**	6	3,622 ± 1,116	–	–	144 ± 23	–	**8.6 ± 2.0**	–
*B. erectum* RRGSC^*d*^	*E. brachyelytri*	**2, 3**	3	–	–		106 ± 17	–	**10.1 ± 2.3**	–
*B. erectum* RRGSC^*e*^	*E. brachyelytri*	**2, 3**	2	–	–	–	tr	–	tr	–
*Elymus* canadensis 04CWR2 Mx^*f*^	*E. canadensis* CWR 34	**1, 2**	1	7,491	–	–	475	–	–	–
Seeds										
*B. erectum* FLCLF	*E. brachyelytri*	**2, 3**	5	–	–	–	87 ± 40	–	9.3 ± 1.6	–
*B. erectum* KRPTD	*E. brachyelytri*	**1, 2, 3**	5	334 ± 109	–	–	**86 ± 16**	–	6.1 ± 0.7	–
*B. erectum* KRPTD	*E. brachyelytri*	**2, 3**	5	–	–	–	**47 ± 10**	–	5.4 ± 1.3	–
*B. erectum* RRGSC	*E. brachyelytri*	**1, 2, 3**	5	3,302 ± 519	–	–	**133 ± 16**	–	**13.0 ± 1.7**	–
*B. erectum* RRGSC	*E. brachyelytri*	**2, 3**	4	–	–	–	**49 ± 4**	–	**8.2 ± 0.7**	–
*El. canadensis* Lincoln Co., TX	*E. canadensis* 119	**1, 2, 3**	1	874	–	–	19	–	5.0	–
*El. canadensis* 04CWR2 Mx^*f*^	*E. canadensis* CWR 34	**1, 2**	1	1,352	–	–	14	–	–	–
*El. canadensis* Throckmorton Co., TX	*E. canadensis* E4815	**1a, 2a, 3**	1	65	1,355	–	tr	22.0	8.1	–
*El. canadensis* PI 1694828^*g*^	*E. canadensis* ECLA-714	nd	1	162	248	–	–	–	5.0	–
Regrowth										
*Lolium pratense*	*E. siegelii* E915	**1a–d, 3b, IDT** ^*h*^	1	8	tr	4,090	–	–	–	197
*L. pratense*	*E. uncinata* E167	**1a–d**	1	9	tr	22,531	–	–	–	–

^*a*^ *n* = number of samples analyzed, nd = not determined, tr = trace levels.

^*b*^ Predicted *Epichloë* chemotypes based on PCR for genes at *LOL, EAS* and *ppzA* loci, and on genome sequences. Alkaloid structures are as in Fig. 1.

^*c*^ SD calculated when more than one sample was analyzed. Entries in bold indicate significant differences (unpaired two-tailed *t* test, *P* < 0.05) between chemotypes for that population and tissue type (excluding outliers); (–) = none detected, tr = trace (detectable but not quantifiable). Amounts of lolines **1a–d** were calculated assuming the response factor of **1d**, and amounts of **3b** were calculated assuming the response factor of **3**. Low levels of **3a** and **3c** were also detected.

^*d*^ Excluding outliers.

^*e*^ Outliers.

^*f*^ Nueva León State, Mexico (56).

^*g*^ PI = Plant introduction number of the USDA-ARS Germplasm Resources Information Network (GRIN).

^*h*^ Genome sequence of the *E. siegelii* isolate indicated *IDT* genes sufficient for biosynthesis of terpendoles, which were not tested.

Among all *LOL+ B. erectum* leaf samples, average levels of **1** ranged from approx 1,350–3,600 µg/g dry wt (Table 2). In some cases, the *LOL*+ and *LOL–* chemotypes differed in levels of the other alkaloids. At KRPTD, *LOL*+ leaf samples had significantly higher levels of **2** (*P* = 0.0019, two-tailed *t* test) and **3** (*P* = 0.0093) compared with *LOL–* samples. In contrast, at RRGSC, *LOL*+ samples and *LOL–* samples had similar levels of **2** and **3** when two outliers were excluded. The *EAS+* and *EAS–* leaf samples from CACAV had similar levels of **1** and **3**, and except for outliers there, the *EAS+* leaves from CACAV had levels of **2** and **3** similar to most leaf samples from other locations.

Comparing levels of alkaloids in leaves and seeds gave contrasting results for the KRPTD and RRGSC populations. Most mature tiller samples had sufficient biomass of both leaves and seeds for analysis, so both paired and unpaired 2-tailed *t* tests were applied with similar results. The paired tests are presented in Table 4. In KRPTD, leaves had significantly higher levels of **1**, **2**, and **3** than did seeds. The difference in **1** was especially large at sixfold higher in leaves than in seeds. In contrast, most RRGSC samples (excluding the two outliers) had levels of **3** in leaves that were lower than in seeds with marginal significance, whereas levels of **1** and **2** were similar in leaves and seeds.

**TABLE 4:** Paired two-tailed *t* tests for differences in alkaloid levels between leaves and seeds at KRPTD and RRGSC populations^*a*^

Statistic	Alkaloid (µg/g dry wt)					
	1		2		3	
	Leaves	Seeds	Leaves	Seeds	Leaves	Seeds
KRPTD (collected 11 Oct 2021)						
Average (µg/g dry wt)	2,028	334	106	69	7.8	6.0
SD	474	109	25	24	1.5	0.9
*n*	5	5	9	9	9	9
*P*	0.0028**		0.0006***		0.0065**	
RRGSC (collected 20 Aug 2022)						
Average (µg/g dry wt)	3,701	3,301	135	118	8.5	12.1
SD	1,229	519	29	39	2.1	2.7
*n*	5	5	6	6	6	6
*P*	0.57		0.247		0.050*	

^*a*^ Asterisks indicate statistical significance. Structures of alkaloids **1**, **2**, and **3** are shown in Fig. 1.

The RRGSC population was analyzed for alkaloid levels in three tissue types (young shoots, mature leaves, and seeds), comparing *LOL*+ and *LOL–* chemotypes for evidence of possible competition between pathways (Table 5). The only direct precursor shared between the pathway to **2** and those to **1** and **3** is *S*-adenosylmethionine, and biosynthesis of **1** and **2** apparently did not compete. In fact, levels of **2** were significantly higher in *LOL*+ compared with *LOL–* shoots (*P* = 0.0008) and seeds (*P* = 0.0001), and trended higher in *LOL* + leaves. In contrast, there was some evidence of competition between pathways to **1** and to **3**, which share l-proline as a precursor. Specifically, young *LOL*+ shoots averaged significantly less **3** than did the *LOL–* shoots (*P* = 0.0157). However, in older leaves and seeds, *LOL*+ averaged slightly more **3** compared *with LOL–*, and the difference was significant in seeds (*P* = 0.0012). These results were consistent with competition between the pathways to **1** and **3** only in young shoots.

**TABLE 5:** Alkaloid data from different plant tissue types in samples from the RRGSC population, comparing endophyte chemotypes^*a*^

Sample	Mating type	Chemotype	Alkaloid (µg/g dry wt)^*b*^		
			1	2	3
**Shoots**					
SC501	*MTB*	**1, 2, 3**	1,354	112	9.4
SC502	*MTB*	**1, 2, 3**	1,810	126	9.5
SC518	*MTB*	**1, 2, 3**	565	144	7.1
SC519	*MTB*	**1, 2, 3**	1,460	124	10.5
SC520	*MTB*	**1, 2, 3**	1,331	108	7.3
SC521	*MTB*	**1, 2, 3**	206	127	5.4
**Mean ± SD**		**1, 2, 3**	**1,121 ± 606**	**123 ± 13**	**8.2 ± 1.9**
SC512	*MTA*	**2, 3**	0	61	8.9
SC513	*MTB*	**2, 3**	0	75	12.5
SC514	*MTB*	**2, 3**	0	76	11.7
SC515	*MTB*	**2, 3**	0	119	8.9
SC516	*MTB*	**2, 3**	0	84	13.0
SC517	*MTB*	**2, 3**	0	88	10.2
SC522	*MTB*	**2, 3**	0	80	12.4
**Mean ± SD**		**2, 3**	**0**	**83 ± 18**	**11.1 ± 1.7**
***t (df)***			**N/A**	**4.5808 (11**)	**2.8518 (11**)
***P***^*c*^			**N/A**	0.0008***	0.0157*
**Leaves**					
SC211	*MTA*	**1, 2, 3**	3,319	156	11.2
SC215	*MTA*	**1, 2, 3**	1,874	100	5.7
SC216	*MTA*	**1, 2, 3**	3,812	138	7.2
SC217	*MTA*	**1, 2, 3**	5,164	157	9.1
SC218	*MTA*	**1, 2, 3**	3,230	150	8.1
SC220	*MTA*	**1, 2, 3**	4,335	162	10.2
**Mean ± SD**		**1, 2, 3**	**3,622 ± 1,116**	**136 ± 23**	**8.6 ± 2.0**
SC201	*MTB*	**2, 3**	0	95	11.3
SC213	*MTB*	**2, 3**	0	126	11.5
SC214	*MTB*	**2, 3**	0	97	7.5
SC209	*MTB*	**2, 3**	0	7 ^*d*^	0.5 ^*d*^
SC219	*MTA*	**2, 3**	0	9 ^*d*^	0.7 ^*d*^
**Mean ± SD**		**2, 3**	**0**	**107 ± 13**	**10.1 ± 2.3**
***t (df)***			**N/A**	**2.1356 (7**)	**1.0301 (7**)
***P***			**N/A**	**0.0701**	**0.34**
**Seeds**					
SC211	*MTA*	**1, 2, 3**	3,763	124	11.2
SC215	*MTA*	**1, 2, 3**	3,620	135	13.9
SC216	*MTA*	**1, 2, 3**	2,448	120	12.5
SC217	*MTA*	**1, 2, 3**	3,461	160	15.5
SC220	*MTA*	**1, 2, 3**	3,217	125	11.9
**Mean ± SD**		**1, 2, 3**	**3,302 ± 519**	**133 ± 16**	**13.0 ± 1.7**
SC202	*MTB*	**2, 3**	0	49	9.2
SC214	*MTB*	**2, 3**	0	56	8.1
SC209	*MTB*	**2, 3**	0	45	7.7
SC219	*MTA*	**2, 3**	0	49	7.8
**Mean ± SD**		**2, 3**	**0**	**49 ± 4**	**8.2 ± 0.7**
***t (df)***			**N/A**	**9.8335 (7**)	**5.2147 (7**)
***P***^*c*^			**N/A**	0.0001***	0.0012**

^*a*^ Statistics are in bold.

^*b*^ Structures of alkaloids **1**, **2**, and **3** are shown in Fig. 1.

^*c*^ Asterisks (*) indicate significance of differences between *LOL*+ and *LOL–* sample sets of the same tissue type based on unpaired two-tailed *t* tests. N/A = not applicable.

^*d*^ Outliers excluded from statistical analyses.

### Alkaloids in other grass-*Epichloë* holobionts

For comparison with the alkaloid profiles in *B. erectum-E. brachyelytri* holobionts, those of other plants with *Epichloë* species were also characterized. Likely chemotypes of *Epichloë siegelii, Epichloë uncinata,* and *Epichloë canadensis* isolates CWR 34 and E4815 were determined from published sequences (GenBank accessions listed in Table 2), whereas those of other *E. canadensis* strains were inferred from PCR results. Alkaloid products predicted from the *LOL* and *EAS* clusters and the *ppzA* gene were quantified in plants with each of these endophytes (but indole-diterpenes were not because, of the endophytes in this study, only *E. siegelii* had an *IDT* cluster). Four qualitatively distinct profiles were obtained from *Elymus canadensis* seeds*,* and two more from *Lolium pratense* tillers with either *E. siegelii* or *E. uncinata* (Table 3).

The alkaloid profile from seeds with *E. canadensis* 119 was qualitatively similar to the common profile in *B. erectum* with **1**, **2**, and **3**, whereas *E. canadensis* CWR 34 produced **1** and **2**, but not **3** (Table 3), in keeping with the alkaloid genes in its sequenced genome (GenBank accession GCA_046865985.1). The profile from seeds with *E. canadensis* E4815 included **1a** and **2a**, along with lower levels of their respective pathway intermediates **1** and **2**. Seeds with *E. canadensis* ECLA-714 also had **1** and **1a**, but contrary to prediction from PCR, they lacked **2**.

The highest reported levels of lolines are from regrowth (after clipping) of *L. pratense* plants symbiotic with *E. uncinata* or *E. siegelii* (54), and we repeated that analysis with the improved quantification in this study (Table 3). Comparing alkaloids in 4-day regrowth tissues of *L. pratense-Epichloë* holobionts to those of *B. erectum* leaves and shoots, the summed levels of **1a–d** in *L. pratense* plants with *E. siegelii* were slightly greater than the highest levels of **1** in *B. erectum*, but the summed levels of lolines (**1a–d**) in *L. pratense* with *E. uncinata* were far higher than levels of 1-aminopyrrolizidines in any *B. erectum* samples, and amounted to 2.25% dry mass of the plant tissue. Almost half of that (10,291 µg/g dry wt) was **1a**, which was measured against pure **1a** standard for reliable quantification.

The *L. pratense* regrowth samples lacked **2** and **3** (Table 3), as predicted from their endophyte genome sequences. Furthermore, in keeping with its *ppzA*-∆R allele, *E. siegelii* produced pyrrolopyrazine-1,4-diones, of which **3b** was by far the most abundant. We estimated the level of **3b** at well over 10-fold higher than was typical of **3** in *B. erectum* samples, but with the caveat that **3b** was quantified assuming the same response factor as **3**.

### Chemotype distribution in genus *Epichloë*

Collating data from a recent survey of 50 sequenced genomes from 28 *Epichloë* species, plus a genome from *Atkinsonella hypoxylon* (1), there were 45 unique combinations of species and predicted chemotypes. Of these, known or predicted chemotypes included six producers of *exo*-1-acetamidopyrrolizidine **1**, 15 producers of loline alkaloid **1a** or **1d**, four producers of chanoclavine **2**, and 13 producers of ergovaline **2a**. Interestingly, none with end-product **1** also had **2a**, and none with end-product **2** also had **1a–d**. Fisher’s exact test with a 3 × 3 contingency table for character states **1**, **1a–d**, and nil for *LOL*, and **2**, **2a**, and nil for *EAS* (Table 6), indicated significance at *P* = 0.0245, suggesting that combinations of alkaloids from the different classes were under selection.

**TABLE 6:** A 3 × 3 contingency table for unique species-by-chemotype combinations predicted from sequenced genomes (1)^*a*^

Cluster and alkaloid^*b*^	*EAS* 2	*EAS* 2a	*EAS* nil	*LOL* totals
*LOL* 1	3	0	3	**6**
*LOL* 1a–d	0	5	10	**15**
*LOL* nil	1	8	15	**24**
*EAS* totals	**4**	**13**	**28**	**45**

^*a*^ Data from 45 genomes from 28 *Epichloë* species and one genome of *Atkinsonella hypoxylon*. Totals are given in bold text. Fisher’s exact test: *P* = 0.0245.

^*b*^ Structures of *LOL* alkaloids **1** and **1a–d**, and *EAS* alkaloids **2** and **2a** are shown in Fig. 1. Absence of an alkaloid class is indicated as “nil”.

### Alkaloid gene relationships and possible horizontal gene transfers

Predictions of pathway end-products have been based on presence or absence of functional genes for each alkaloid pathway (1). Termination at **1** is predicted for strains lacking functional *lolO* but possessing functional genes for the first five steps in the 1-aminpyrrolizidine pathway, and termination at **2** is predicted for strains lacking functional *easD* but possessing functional genes for the first four steps in the ergot alkaloid pathway (Fig. 1).

**Fig 2:**
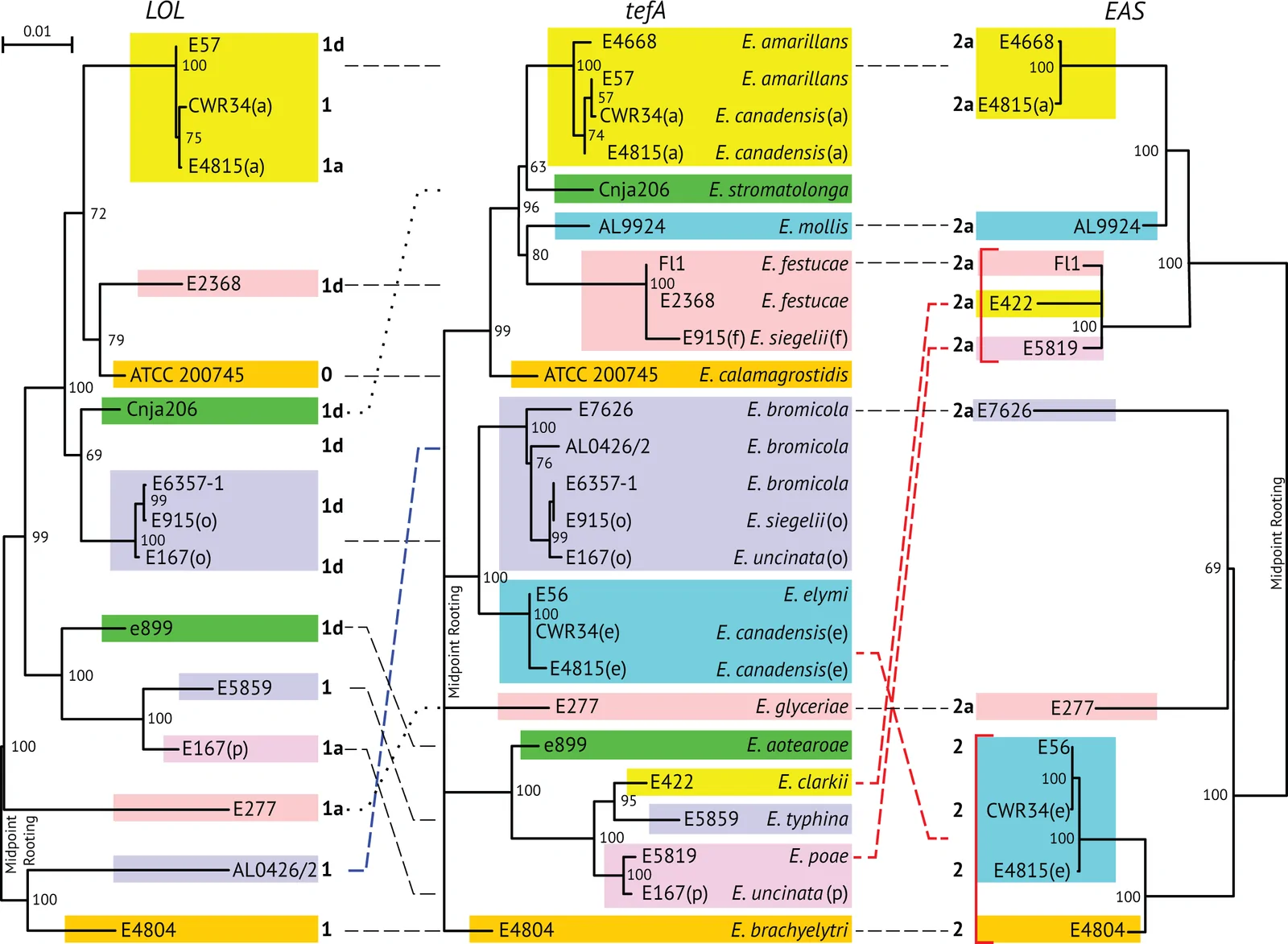
Tanglegrams comparing phylogenies of early-pathway *LOL* and *EAS* genes and the housekeeping gene *tefA*. The *lolC, lolF,* and *lolE* genes were aligned and concatenated for the *LOL* phylogeny (left), and the *dmaW, easF, easE,* and *easC* genes were aligned and concatenated for the *EAS* phylogeny (right), and trees were inferred with PhyML. Known or predicted alkaloid products are indicated as 0 (no alkaloid of that class), 1, 1a, 1d, 2, or 2a. Branch lengths are proportional to estimated substitutions per site. Bootstrap branch support values (100 replications) are indicated at the nodes. Branches with less than 50% bootstrap support have been collapsed into polytomies. Colored boxes indicate clades that include haploid species plus genes in interspecific hybrids that were acquired from their *E. amarillans* (a), *E. elymi* (e), *E. festucae* (f), or *E. bromicola* (o) ancestors. Dashed lines connect isolates or clades between phylogenies, and dotted lines indicate topological incongruencies attributable to branches with low bootstrap support. A thick blue dashed line indicates a phylogenetic conflict between *LOL* and *tefA* phylogenies that is indicative of a trans-species polymorphism extant in *E. bromicola* (1). Thick red dashed lines and square brackets indicate incongruencies between the *EAS* and *tefA* phylogenies that suggest possible horizontal gene transfers (HGT).

Relationships of species and strains with different chemotypes were investigated by phylogenetic analysis of concatenated gene sequences (including introns) for *lolC, lolF,* and *lolE* representing the *LOL* clusters, *dmaW, easF, easE,* and *easC* representing the *EAS* clusters, and *tefA,* a housekeeping gene (Fig. 2). With few exceptions, the *LOL* and *EAS* trees were largely, but not entirely, congruent with *tefA*.

In the *LOL* phylogeny (Fig. 2), the only clear deviation from the *tefA* tree topology was the deep branching of *E. bromicola* AL0426/2 relative to its conspecific isolate E6357-1, thus constituting trans-species polymorphism (1). Conceivably, this polymorphism originated from horizontal gene transfer (HGT), but the tree groups AL0426/2 and *E. brachyelytri LOL* genes close to the midpoint root, so it is also plausible that lineage sorting in an ancestral *Epichloë* species was responsible. Aside from that relationship, the *LOL* phylogeny did not group strains according to their pathway end-products, indicating independent origins of chemotypes that terminate this pathway at **1**.

Comparison of *EAS* and *tefA* phylogenies (Fig. 2) indicated conflicts suggesting at least two instances of HGT. One HGT event (or possibly two) was indicated by the well-supported clade in the *EAS* tree that included *E. festucae* Fl1, *E. clarkii* E422, and *E. poae* E5819. The position of this clade was consistent with the position of *E. festucae* but dramatically conflicted with the positions of *E. clarkii* and *E. poae* in the *tefA* tree. Another HGT event was suggested by the grouping of *E. brachyelytri* with *E. elymi* and related *E. canadensis EAS* clusters, thereby conflicting with the *tefA* tree, which instead grouped *E. elymi* with *E. bromicola* as sister clades. Therefore, the shared characteristic of *E. brachyelytri* and *E. elymi* (and *E. elymi-*derived) genomes lacking functional genes for steps downstream of **2** may have been due to HGT of an ancestral *EAS* cluster with *dmaW, easF, easE,* and *easC* as the only functional *EAS* genes. Alternatively, the basal polytomy in the *tefA* tree presents lineage sorting as a plausible explanation for this relationship of *EAS* clusters.

**Fig 3:**
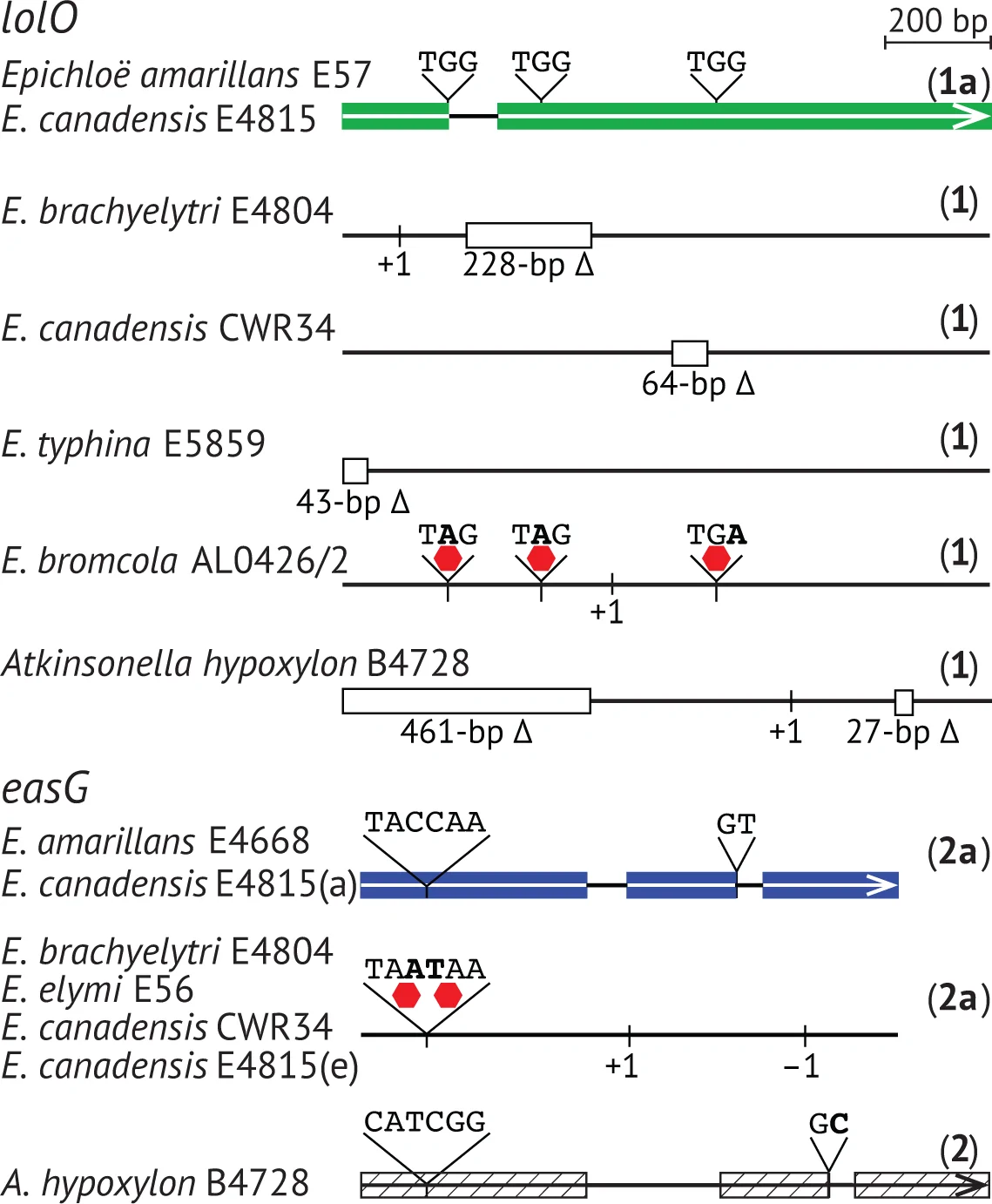
Functional *lolO* and *easG* genes in *Epichloë amarillans* and *Epichloë canadensis* strain E4815 compared with nonfunctional orthologs in other species and strains. Colored boxes indicate coding sequences of the functional genes, and arrows indicate directions of transcription. In the nonfunctional orthologs, frameshift indels are indicated by +1 or –1, larger deletions are indicated by open boxes labeled with deletion lengths, and nucleotide substitutions causing premature translation stops (red hexagons) are indicated in bold. The *easG* map for *A. hypoxylon* is hatched to indicate possible functionality because the intron with a 5′-GC junction might be spliced. Predicted pathway end products of each strain are indicated as **1**, **1a**, **2**, and **2a** in parentheses. Inactivation of *lolO* results in termination of the 1-aminopyrrolizidine pathway at **1**, whereas strains with inactivated copies of *easG* have also lost all or part of the *easD* gene (not shown), causing termination of the ergot alkaloid pathway at **2**. Inactivating mutations in *lolO* differ in each strain, whereas inactivating mutations in *lolG* are similar in three *Epichloë* species.

Termination of the 1-aminopyrrolizidine pathway at **1** instead of lolines was attributable to defects in *lolO,* and different inactivating deletions and mutations in *lolO* were identified in *E. brachyelytri* E4804, *E. bromicola* AL0426-2, *E. canadensis* CWR 34, *E. typhina* E5859, and *A. hypoxylon* B2748 (Fig. 3). The *E. brachyelytri LOL* locus retained fragments of *lolM* (GenBank accession JF800659.1 positions 826–1084), *E. canadensis* CWR 34 had a fragment of *lolP* (accession PP187338.1), and *Epichloë poae* E5819 had all *LOL* genes with only *lolO* sequence indicating an inactivating deletion (accessions PP187346–PP187346; and see below).

In the ergot alkaloid pathway, the step after **2** is determined by *easD*. Results of BLASTn searches (not shown) indicated only fragments of *easD* in *E. brachyelytri* E4804 and *A. hypoxylon* B2748 (NCBI accession GCA_000729835.1), and its absence in *E. elymi* E56 (GCA_000315335.1) and *E. canadensis* CWR 34 (GCA_046865985.1). Interestingly, these genomes also had complete but inactive copies of *easG*, in which the three *Epichloë* strains shared mutations that resulted in tandem premature stop codons and two frameshifts (Fig. 3).

Compared with *E. brachyelytri, A. hypoxylon* also had an *EAS* cluster with apparently functional *dmaW, easF, easE,* and *easC*, a fragment of *easD,* and an *easG* gene that may or may not have been inactivated by a mutation at the second position of intron 2 (Fig. 3). All *EAS* genes of *A. hypoxylon* were distantly related to those of the *Epichloë* species (2), so its predicted pathway termination at **2** is a convergent trait in the two genera.

**Fig 4:**
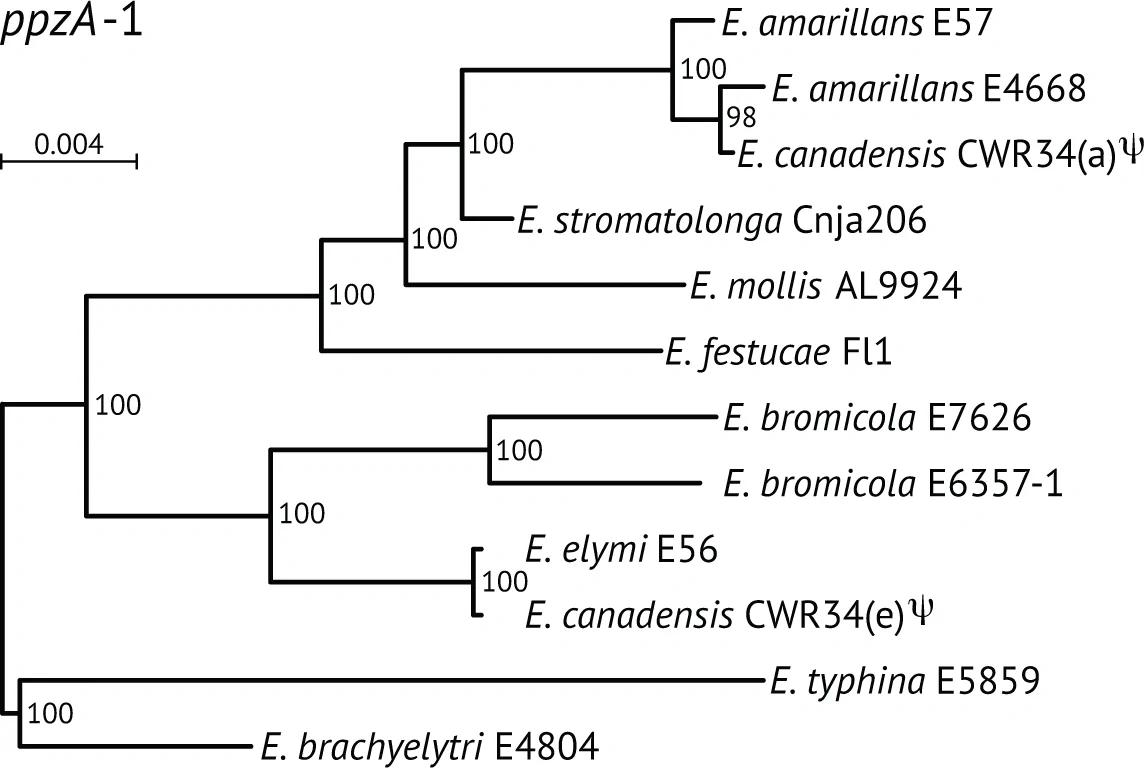
Phylogeny of *ppzA*-1 genes. Trees were inferred with PhyML. Both genes of CWR 34 are designated as pseudogenes (ψ) because they had inactivating frameshift mutations. All other *ppzA*-1 genes appeared potentially functional. Branch lengths are proportional to estimated substitutions per site (scale bar). Bootstrap branch support (100 replications) is indicated at the nodes.

The phylogeny of *ppzA*-1 genes that have the R domain for biosynthesis of **3** matched the topology of the *tefA* gene with high statistical support of all branches (Fig. 4). No *ppzA*-∆R alleles, such as that of *E. siegelii*, were included in this analysis because of their history of trans-species polymorphism and intragenic recombination (9). Both *ppzA-*1 genes from *E. canadensis* CWR 34 had frameshift mutations, explaining the absence of peramine in the plant with that endophyte (Table 3). However, the sequences of their counterparts in *E. canadensis* E4815, of which at least one should be responsible for its production of **3**, were not included because they failed to assemble completely from the genome sequence data.

In summary, genetic and phylogenetic evidence indicated separate evolutionary pathways to *Epichloë* strains with the novel **1 + 2** (+ **3**) chemotype, whereby the *lolO* genes were convergently inactivated in several lineages, but the associated *EAS* cluster variant appears to have evolved only once in genus *Epichloë* by gene losses and inactivation, and may have been transferred between the *E. brachyelytri* and *E. elymi* lineages in an HGT event.

## DISCUSSION

### A novel profile of alkaloids produced by *Epichloë* species

In this survey of the woodland cool-season grass *B. erectum,* we identified **1**, **2,** and **3** (Fig. 1) as the main alkaloids produced by its seed-transmitted fungal symbiont *E. brachyelytri*, which is therefore the first fungal species shown to produce both **1** and **2** as pathway end-products. In 16 other known *Epichloë* species, **1** is an intermediate in the pathway to the anti-insect lolines (1, 7), and, in many fungal species, **2** is an early intermediate to the lysergic acid amides or ergopeptines such as **2a,** which, unlike **2** (57), are notoriously toxic to mammals (reviewed in references 2, 58). In addition to *E. brachyelytri*, other *Epichloë* species and strains, as well as *A. hypoxylon*, were identified with confirmed or predicted pathway termination at **1**, **2**, or both, and we identified cases of convergent evolution of the **1 + 2** chemotype involving gene losses, pseudogenization, putative HGT, and interspecific hybridization. Altogether, the results of this study are consistent with fitness benefits and possible synergy of **1** and **2**, which we suggest are also likely to protect against invertebrate herbivores, but perhaps with different activity spectra compared with the more complex lolines and ergopeptines.

### The metabolite analytical platform

The approach adapted for this study involves one simple extraction of each sample, followed by its comprehensive, high-sensitivity, and high-resolution analysis. The Agilent 6546 platform for UHPLC-LC-qTOF analysis delivers sub-ppm mass accuracy (<0.8 ppm RMS) and high resolving power, enabling confident elemental formula assignment and discrimination of structurally related or isobaric alkaloids produced by symbiotic microbes within complex plant matrices. The sensitivity of the Agilent 6546 instrument (signal-to-noise ratio >500:1 in MS at 1 pg on-column) supports reliable quantification of analytes present at low levels (µg/g dry wt), depending on matrix and ionization efficiency. Acquisition of full-scan high-resolution data together with MS/MS fragmentation enables simultaneous targeted quantification and structural verification through diagnostic fragmentation patterns. The wide dynamic range of the platform permits direct comparison of major and minor alkaloids within individual samples, while preserving a comprehensive accurate-mass data set that can be retrospectively interrogated for additional known or previously uncharacterized metabolites. Collectively, this LC-qTOF framework provides a robust, sensitive, and discovery-enabled platform for quantitative chemotype profiling of environmental samples (as in this study), as well as for laboratory studies of biosynthetic pathways.

### Qualitative and quantitative variation of alkaloids in *B. erectum*

The most abundant alkaloid in plants with *LOL+ E. brachyelytri* was **1**, and its biosynthesis may exact an ecologically significant metabolic cost, as suggested by its +/– polymorphism and evidence of competition between pathways to **1** and **3** in young shoots of *B. erectum*.

We observed moderate-to-high levels of **1** and **2** in most *B. erectum* samples, and we expect, therefore, that loss of genes for either alkaloid can be advantageous in environments where the metabolic cost of producing them is insufficiently offset by herbivore pressure, so we hypothesized that its biosynthesis exacts a substantial metabolic load and competes with other biosynthetic processes such as the pathway to **3**, with which it shares l-proline as a precursor. We quantified **1**, **2**, and **3** in sets of plants with and without **1** to test for competition between the respective pathways. Although we saw no trend indicative of such competition in seeds and leaves from mature tillers, we did see a significant difference in young shoots that was consistent with competition for biosynthesis of **1** and **3** (Table 5).

Overall, endophyte growth and metabolism in growing plant tissue (43, 59) certainly constitute a metabolic cost to the host that is partly related to relative endophyte biomass. Typical estimates of *Epichloë* biomass are 1.5% or less of host biomass (60, 61). However, the fungal alkaloids add to that cost, particularly for 1-aminopyrrolizidines (**1** and the lolines **1a–d**) that can accumulate to levels comparable to endophyte biomass (54). Therefore, production of alkaloids **1** and **2** may be sufficiently costly to select for endophyte variants that have lost *LOL* or *EAS*, depending on the balance between metabolic resources and pressure from herbivores that may be affected by those alkaloids.

In addition to gene losses and inactivation, other mechanisms also generate variation in alkaloid profiles as evidenced by four outlier samples with exceptionally low levels of **2**, **3**, or both. Possible causes for such outliers could be genetic or epigenetic, manifestations of host control, or a combination of those factors. In the two outlier plants at RRGSC, leaves had low levels of **2** and **3**, but seeds had levels similar to those of other plants, suggesting that their leaves may have had low endophyte biomass. Analogously, it is known that the *L. arundinaceum* endophyte *E. coenophiala* largely fails to colonize leaf blades in some common ecotypes and cultivars but is nevertheless present in leaf sheaths and seeds (60, 62), where most of alkaloid **2a** accumulates (10, 63, 64). However, a biomass-based explanation seems less likely for *B. erectum* samples with exceptionally low levels of just one alkaloid (**2** or **3**) but typical levels of the others (Tables 3 and 5). More likely, these outliers have regulatory or functional mutations, or epigenetic changes that affect those respective alkaloid pathways.

The existence of *E. brachyelytri* strains lacking *LOL* or *EAS* genes, as well as the outlier we identified with only trace amounts of **3**, demonstrate that none of these alkaloids alone is required for survival of the holobionts, and indeed the presence of 6%–58% endophyte-free plants in our surveyed locations demonstrates that the epichloid alkaloids are not essential for plant survival. Perhaps, E– plants and the mutant chemotypes are cheaters that benefit from herbivore deterrence from neighboring plants with **1**, **2,** and **3**. At least with respect to alkaloid **1**, we do not favor the cheating hypothesis as a generality considering that only *LOL–* endophytes were identified in the FLCLF population. Also, the RRGSC population included chemotypes with and without **1**, as well as E– plants, such that only 24% of the plants possessed alkaloid **1**, which seems inadequate for this alkaloid to defend the overall population. Therefore, the explanation we favor for the observed chemotypic variation is that at some locations and times, the pressure and specificity of herbivorous invertebrates is insufficient to counterbalance the metabolic cost of producing **1**. A similar scenario may explain the observed +/– variation in **2** at CACAV, considering the high levels of **2** produced by most *EAS*+ strains of *E. brachyelytri* there and elsewhere.

### Quantitative comparisons of alkaloids in different species

The levels of **1** that we measured in *B. erectum* leaves were in the high range compared with levels of lolines (**1a–d**) in mature leaves of several other grass-*Epichloë* symbiota (reviewed in references 29, 65). They were similar to total levels of lolines in mature leaves of *L. arundinaceum* with *E. coenophiala, L. pratense* with *E. uncinata* (54), and *Festuca versuta* with *Epichloë* sp. (66). In this study, we also compared alkaloid levels in young shoots of *B. erectum* to young regrowth (after clipping) of *L. pratense* with symbiotic *E. uncinata* or *E. siegelii* (Table 3), having previously established that lolines in these *L. pratense* holobionts accumulate in regrowth to significantly higher levels than in mature leaves (54). Here, we confirmed high levels of lolines (**1a–d**) in *L. pratense* with *E. uncinata*, but young shoots of *B. erectum* did not have exceptionally high levels of **1**. Instead, we found comparable or lower levels of **1** in young shoots than in mature *B. erectum* leaves while also finding levels of **2** and **3** to be similar between the two tissues. Thus, the relative levels of 1-aminopyrrolizidines in young versus mature *B. erectum* tissues do not mirror that in *L. pratense*. A caveat is that these two plant species differ in their development whereby *L. pratense* has both vegetative and flowering tillers, whereas *B. erectum* has only the latter. (Vegetative tillers of *L. pratense* include a pseudostem consisting of the sheaths of fully expanded leaves wrapped around each other and younger developing leaves in a “whorl,” whereas *B. erectum* shoots consist of leaves and elongating true stems.) Furthermore, contrasting distributions of **1** versus **2** and **3** in *B. erectum* tissues (Table 4) suggest that relative endophyte biomass is not necessarily the determining factor for alkaloid levels. Pathway regulation and precursor availability in the plant intercellular spaces are alternative, but not mutually exclusive, explanations for different levels of alkaloids in different tissues.

Historically, chanoclavine (**2**) was first identified as an early intermediate in ergot alkaloid biosynthesis (67), whereas ergopeptines such as **2a** were of interest as some of the most potent ergot alkaloids in mammalian systems (11). The pathway from **2** to **2a** requires activities of seven gene products, some of which catalyze multiple steps (reviewed in reference 2) (Fig. 1). Although the ergot alkaloid pathways include some spur products, and intermediates can also accumulate (68), it is remarkable that we find levels of **2** in *B. erectum-E. brachyelytri* holobionts that are 12–44-fold higher than typical levels of **2a** in toxic grass-*Epichloë* symbiota (69).

The levels of peramine (**3**) in *B. erectum* samples (Tables 3 and 4) were comparable to levels reported in many other grass-*Epichloë* systems which, however, are highly variable. In mature vegetative tissues, peramine has been reported at concentrations of approx 1–56 µg/g dry wt (55, 70–73), but have ranged as high as 132–347 µg/g dry wt in some *Epichloë-*symbiotic grasses (9, 72). Interestingly, we confirmed very high levels of the related alkaloid **3b** in *L. pratense* with *E. siegelii* (9), although precise quantitation would require a pure **3b** standard.

### Evolution of early-terminating alkaloid pathways

In addition to *E. brachyelytri*, we found chemotypes with **1 + 2** (sometimes including **3**) in two other *Epichloë* species, and this profile is predicted from the *A. hypoxylon* genome sequence as well (1, 56, 74). Comparing their *lolO* genes, we identified independent mutations responsible for their pathway termination at **1** (Fig. 3). The situation is more complicated for *EAS*, for which pathway termination at **2** was attributable to two independent variants (one shared by *Epichloë* species and the other in *A. hypoxylon*), plus a likely HGT between *Epichloë* species. Thus, the apparent origins of **1 + 2** chemotypes are independent mutations of *LOL* in *A. hypoxylon, E. brachyelytri,* and *E. amarillans* lineages, independent mutations of *EAS* in *A. hypoxylon,* and an *Epichloë* lineage, either lineage sorting or HGT of the variant *EAS* cluster in *E. brachyelytri* and *E. elymi* (or their ancestors), and hybridizations of *E. amarillans* and *E. elymi* to generate strains of *E. canadensis* with that chemotype. Further chemotypic variation has involved loss of *LOL* or *EAS* in *E. brachyelytri* lineages, and independent hybridizations of other *E. amarillans* chemotypes (**1a**, and **1a + 2a**) with *E. elymi*, thereby generating the various *E. canadensis* chemotypes reported previously (56).

Interestingly, in our survey of fungal species with sequenced genomes (1), the frequencies of known and predicted chemotypes significantly deviated from expectations (Table 6). Chemotypes with **1 + 2** apparently arose independently in three fungal species, but chemotypes with **1 + 2a** or **1a–d + 2** have not been found. The pattern may result from synergism between particular alkaloid, species such as **1** and **2**, negative effects of the missing alkaloid combinations, or both, which in turn may explain the convergent evolution of **1 + 2** chemotypes in *E. brachyelytri, E. canadensis,* and *A. hypoxylon*.

### Conclusions

The genus *Epichloë* exhibits a variety of mechanisms for genetic and chemotypic diversification. Most but not all haploid species have documented sexual cycles, but in many, including *E. brachyelytri*, it is very rarely observed, and clonal propagation in host seeds predominates (1, 26). Furthermore, this fungal genus includes many polyploid interspecific hybrids that are apparently asexual (26, 30). Hybridization may combine (pyramid) alkaloid gene clusters and may also account for HGT between haploid species such as the possible transfer of *EAS* between *E. brachyelytri* and *E. elymi* (or their respective ancestors). These processes, together with mutations and deletions of individual genes and whole gene clusters, illustrate highly dynamic evolution of *E. brachyelytri* chemotypes that is likely to be adaptive for the environmental and temporal heterogeneity experienced by its host grass.

## MATERIALS AND METHODS

### Biological and chemical materials

Locations and designations of the six sampled *B. erectum* populations on a 171 km transect in Kentucky are given in Table 1. Shoots with one fully expanded leaf were sampled from the KYARB and RRGSC populations in mid-Spring, immature tillers prior to inflorescence emergence were sampled from CACAV in late Spring, and mature and healthy tillers with seeds were sampled from the FLCLF, KRPTD, MACAV, and RRGSC populations in Summer and early Autumn. For storage prior to alkaloid analysis, tissues were freeze-dried and kept dry at room temp.

Seeds of *El. canadensis* without endophyte (E–) or with each of two chemotypes of *E. canadensis* were provided by Dr. Nikki D. Charlton (Noble Research Institute, Ardmore, OK) and planted on the grounds of Leestown Middle School in Lexington, Kentucky, then seeds were harvested in the second year after planting.

Plants of *L. pratense* with endophyte *E. siegelii* or *E. uncinata* were maintained in the greenhouse in Lexington, Kentucky (54). Plants were clipped, then after 4 days, the regrowth was harvested for alkaloid analysis.

### PCR screen for alkaloid biosynthesis and other genes

DNA was isolated from stem sections of *B. erectum* plants from each location with the DNeasy 96 Plant Kit (Qiagen, Valencia, CA, USA) according to manufacturer’s recommendations. PCR tests with primers (Table 7) and protocols for multiplex and single-target PCR as described by Charlton et al. (75) were conducted for mating-type genes *mtAC* (MK992971.1) and *mtBA* (MK992932.1), and genes for the determinant steps in the alkaloid pathways: *dmaW* (GenBank accession JN177507.1), *lolC* (JF800661.1), and *idtG* (= *ltmG*; JN613320.1), and for the *ppzA*-1 (= *perA*; JN613323.1) T2 and R (reductase) domains.

**TABLE 7:** PCR primers used to screen genes^*a*^

Gene/domain	Primer name	Sequence (5′–3′)	Pos.	Or.
*dmaW*	dmaW-F4	GTG TAC TTT ACT GTG TTC GGC ATG	1051	F
*dmaW*	dmaW-6R	GTG GAG ATA CAC ACT TAA ATA TGG C	1333	R
*lolC*	lolC-5b	TCT AAA CTT GAC GCA GTT CGG C	334	F
*lolC*	lolC-3a	GGT CTA GTA TTA CGT TGC CAG GG	775	R
*idtG* (*= ltmG*)	idtG-F	GAG CTT GAG AAG CTT ACG AAT CC	493	F
*idtG*	idtG-R	GGG CAA TGG AGC GAT TCT CTC	605	R
*ppzA* (*= perA*)/T2	per T2-F	TCT TCA GGC ATC GCA GGA AC	6378	F
*ppzA*/T2	per T2-R	TCG GCC ACC TCC AGC CTG ATG	6977	R
*ppzA*/reductase	per red F2	GAG ATC AGT TCG CAG TTG TCA G	7739	F
*ppzA*/reductase	per red R	CTA GCC TCC AGA TCT TGT GAA AG	8325	R
*mtAC*	mtAC-F	CAA TGG TGG TCA CCT GAG AAG	–174	F
*mtAC*	mtAC-R	CGG TCT CAT TCT TCC AGA GAG AGG	611	R
*mtBA*	mtBA-F2	ATC AGT TGA GGG CGA TTT GG	44	F
*mtBA*	mtBA-R2	AGG CTT GCT TGA CTC TAT CCG C	662	R

^*a*^ Pos. = position (bp, relative to start codon), Or. = orientation, F = forward, R = reverse.

### Alkaloid extraction

Reagents used for the extraction solvent were methanol (>99.9% pure for LC-MS; Honeywell Burdick & Jackson, Muskegon, MI), water purified by ion-exchange and reverse osmosis to 18.2 MΩ·cm resistivity and total organic carbon <5 ppb, and formic acid at >99.0% purity (Optima LC/MS Grade, Thermo Fisher Scientific, Pittsburgh, PA). Extraction solution was 80:20 v/v methanol and water with 0.1% formic acid. The *B. erectum* leaf or shoot samples were flash frozen, ground to fine powder in liquid nitrogen, and freeze-dried. For dry seeds, a measured amount (usually 30 mg) was weighed into 2-mL microtubes (Eppendorf Safe-Lock 2.0, from Thermo Fisher Scientific), and to each tube was added two 3-mm stainless-steel beads. These were then agitated with a Genogrinder 2010 (Spex Sample Prep, Metuchen, NJ) at 1,700 strokes/min for 5–7 min until the tissue was finely powdered. Samples were extracted by the protocol of Vassiliadis et al. (76) with modifications. A measured amount of sample (usually 25 mg) was added to 500 µL of extraction solution with 2 ng/mL analytical grade quinoline (Sigma Aldrich, St. Louis, MO) as internal standard. The samples were vortexed for 10 min and sonicated for 5 min (Digital Ultrasonic Cleaner, Vevor, Rancho Cucamonga, CA), then centrifuged for 10 min at 16,000 × *g* at room temperature. The supernatant was saved, and the pellet was reextracted with another 500 µL of extraction solution. The supernatants were pooled and centrifuged to clarity, then transferred to clear glass vials for UHPLC-MS analysis.

### Chromatography

Samples were chromatographed using an Agilent 1290 Infinity II UHPLC coupled to an Agilent 6546 time-of-flight mass spectrometer (Agilent Technologies Santa Clara, CA). A 150 × 4.6 mm Luna Omega 3 µM polar C18 column was used for reverse phase chromatography with water containing 0.1% formic acid as mobile phase A and acetonitrile containing 0.1% FA as mobile phase B. The following gradient was used for separation with a flow rate of 0.5 mL/min: 0–5.5 min (10% B), 5.5–11.50 min (10%–100% B), and 11.60–16 min (100%–10% B). Mass spectrometry (MS) was in positive mode with the following parameters: gas temp, 300 °C; drying gas flow rate, 12 L/min; nebulizer, 40 psi; sheath gas temp, 250°C; sheath gas flow, 12 L/min; fragmentor voltage, 100 V; skimmer voltage, 65 V; nozzle voltage, 0 V; capillary voltage, 3,500 V. Data were analyzed using MassHunter Qualitative (v. 12.0) and Quantitative (v. 11.0) software.

### Alkaloid quantification

As standards, pure **1** was synthesized in the laboratory of Dr. Robert B. Grossman (University of Kentucky) for a previous study (77), **1d** was purified from *Epichloë*-infected tall fescue seeds (21), pure **2** was generously provided by Dr. Daniel G. Panaccione (West Virginia University), and pure **2a** and **3** were synthesized by BenchChem (Austin, TX) and generously provided by Dr. Pilar Catalán (University of Zaragoza, Spain). Identities of the alkaloids in plant materials were verified by high-resolution m/z determination for the parent ions, and comparisons to published MS/MS profiles (9, 78).

For standard curves, the pure alkaloids were diluted in extraction solution (see above) containing quinoline at 2 ng/mL. Each standard curve was based on response ratios of analyte to quinoline for 5–7 serial twofold dilutions over which the values fit a linear or quadratic regression through the origin at *R*^2^ > 0.997.

Each sample was analyzed undiluted, at a 1:100 dilution, and sometimes at higher dilutions to ensure that response ratios fell within the standard curve for the analyte. Dilutions were in extraction solution (see above) with quinoline (2 ng/mL). Concentrations of **1b–d** were estimated based on the standard curve for **1a**, and concentrations of **3a–d** were estimated based on the standard curve for **3**. For all analytes in each sample, the correction factor for extraction efficiency was the response value for quinoline in the 1:100 diluted sample, divided by 0.99 × the response value for quinoline in the undiluted sample.

### Genome annotations and phylogenetics

Genome sequences published on NCBI were downloaded and analyzed by local BLASTn, and alkaloid genes were manually annotated based on known orthologs in *Epichloë* species. Accession numbers are listed in Table 2. The annotated genes for each cluster were aligned using ClustalW, adjusted manually as needed, and concatenated according to their alkaloid clusters to produce an alignment of *lolC, lolF,* and *lolE* for *LOL*, an alignment of *dmaW, easF, easE,* and *easC* for *EAS*, an alignment of *ppzA*-1, and an alignment of *tefA* encoding the housekeeping protein translation elongation factor 1-α. All alignments included gene coding sequences and introns.

Phylogenetic analyses utilized PhyML implemented on the NGphylogeny.fr website (79) for maximum likelihood inference with the model GTR + Γ (four categories and estimated proportions of invariant sites), empirical base frequencies, and SPR topology searches. Branch supports were estimated by 100 bootstrap replications. Tanglegrams were produced by visual comparisons of the gene trees.

### Statistics

Quantitative comparisons between chemotypes and between tissues were analyzed by two-tailed *t* tests. These were paired tests when leaves and seeds were analyzed from the same tillers, or else they were unpaired tests. Samples with only trace levels of one or more of the alkaloids, with Z-scores < –1.65 (cumulative probability < 0.05), were reported as outliers and excluded from statistical analyses.

## Data Availability

The high resolution UHPLC-MS and MS/MS spectra of **1**, **1d**, **2**, **2a,** and **3** in standards, and for **1a**, ergovalinine (the *8S* isomer of **2a**), and pyrrolizidines **3a** and **3b** in plant samples, alkaloid levels for each sample analyzed, and sequence alignments for the phylogenetic analyses are available at https://doi.org/10.5281/zenodo.19547081. All genome sequences used in this study are available from NCBI, and their accessions are listed at https://doi.org/10.6084/m9.figshare.28009748.v1. DNA sequence accession numbers are listed in Table 2.
